# The main effect and gene-environment interaction effect of the *ADCYAP1R1* polymorphism rs2267735 on the course of posttraumatic stress disorder symptoms—A longitudinal analysis

**DOI:** 10.3389/fpsyt.2022.1032837

**Published:** 2022-10-28

**Authors:** Jingyi Zhang, Gen Li, Haibo Yang, Chengqi Cao, Ruojiao Fang, Ping Liu, Shu Luo, Guangyi Zhao, Yingqian Zhang, Kunlin Zhang, Li Wang

**Affiliations:** ^1^Laboratory for Traumatic Stress Studies and Center for Genetics and BioMedical Informatics Research, CAS Key Laboratory of Mental Health, Institute of Psychology, Chinese Academy of Sciences, Beijing, China; ^2^Department of Psychology, University of Chinese Academy of Sciences, Beijing, China; ^3^Academy of Psychology and Behavior, Tianjin Normal University, Tianjin, China; ^4^People’s Hospital of Deyang City, Deyang, Sichuan, China

**Keywords:** *ADCYAP1R1*, posttraumatic stress disorder, SNP, gene-environment interaction, longitudinal analysis

## Abstract

**Background:**

Many studies have been performed to investigate the association between the *ADCYAP1R1* polymorphism rs2267735 and posttraumatic stress disorder (PTSD), but the results have been inconsistent, and the way in which this gene affects the course of PTSD has not been widely investigated. Thus, a longitudinal study of the course (development trajectory) of PTSD is needed.

**Methods:**

In this study, we performed a longitudinal analysis of rs2267735 in 1017 young, trauma-exposed Chinese people (549 females and 468 males, ranging from 7 to 11 years old). At four time points after trauma exposure (2.5, 3.5, 4.5, and 5.5 years), we measured PTSD symptoms with the University of California, Los Angeles PTSD Reaction Index (PTSD-RI) for DSM-IV (Child Version). We employed a latent growth model (LGM) for the longitudinal data to test the association between rs2267735 (main and gene-environment interaction effects) and the course of PTSD symptoms.

**Results:**

The results of LGM showed that the gene-environment interaction (rs2267735 × trauma exposure) effects were associated with PTSD symptoms in girls at 2.5 years (β = –0.291 and *P* = 0.013 for LGM intercept). The gene-environment interaction (rs2267735 × trauma exposure) effect was also correlated with PTSD symptoms in girls at 3.5 and 4.5 years (β = –0.264 and *P* = 0.005; β = –0.217 and *P* = 0.013).

**Conclusion:**

Our study revealed that the gene-environment interaction of the *ADCYAP1R1* polymorphism rs2267735 is associated with PTSD symptoms in girls at 2.5 years and that the effects may be stable over time and not related to the PTSD symptom recovery rate. This is the first study to detect the how the *ADCYAP1R1* gene affects the course of PTSD after trauma exposure in a longitudinal view.

## Introduction

### Posttraumatic stress disorder and its negative consequences

Posttraumatic stress disorder (PTSD) is a mental disorder that often occurs after exposure to traumatic events. PTSD not only has the potential to cause individual psychological and behavioral changes but also usually causes the loss of certain social functions (1). It has been reported that 50–60% of American adults experience at least one traumatic event in their lifetime, and 7–30% will develop PTSD (2). The natural disasters such as hurricanes, earthquake and fires usually caused generalized PTSD and some other health problems (3). A study reported the children showed more serious PTSD symptom in the 21 months after they were exposed to Hurricane Andrew (4).

Compared to the adults, young children who have survived disasters appear more emotional and physical vulnerability (5) and they are diagnosed PTSD at a high rate (6). Additionally, the children with PTSD would suffer serious functional impairment of social and education (7, 8), affect their coping style, adaptation and recovery of disaster in the future (9) and increase risk of somatic problems, depression, anxiety, and suicide (10). However, the childhood and adolescence are important period for establishing long-term emotional health. Thus, it is necessary and meaningful to characterize the PTSD trajectories of children and adolescents to identify high-risk individuals and prove effective intervention (11).

### *ADCYAP1R1* affects posttraumatic stress disorder

Genetic factors are closely related to the occurrence and development of PTSD (12, 13). A study found that genetic factors can explain more than 70% of the PTSD risk variation in young women (12). Duncan et al. (14) found that the heritability of PTSD in the female population is 29%. Further study through a large-scale genome-wide study (GWAS) indicated that the heritability is 5–20%, varying by sex (15).

Pituitary adenylate cyclase activating polypeptide (PACAP) is a neuropeptide and hormone (16), and it is widely distributed in the hypothalamus and amygdala (17, 18). PACAP can help individuals regulate stress or fear effectively and plays an important role in the stress response (19). Moreover, the selective PACAP receptor PAC1 (encoded by the *ADCYAP1R1* gene) not only affects an individual’s stress axes in the long term and influences the stress response (20) but also participates in continuous stress and regulates physiological or psychological responses during stress (21).

In addition, many studies have shown that PTSD is associated with *ADCYAP1R1* and its gene-environment interactions. Ressler et al. (22) made a groundbreaking discovery that *ADCYAP1R1* contributed to the severity of PTSD symptoms in severely traumatized females. Lind et al. (23) showed through a meta-analysis that the C allele of rs2267735 of *ADCYAP1R1* is significantly associated with an increased risk of PTSD in women. Almli et al. (24) found that for both main and gene-environment effects in females, the C allele of rs2267735 was more correlated with PTSD symptom severity than the G allele.

### The necessity of a longitudinal study: Inconsistent results and how the gene affects the course of posttraumatic stress disorder symptoms

According to meta-analytic studies, the relationship between the *ADCYAP1R1* polymorphism and PTSD is controversial (23). Many studies have shown a significant association and suggest that the C allele confers an increased risk of PTSD development (24). However, some studies did not replicate this association (25) and even indicated that *ADCYAP1R1* could not predict PTSD (26, 27). The reason why the results are inconsistent may be related to their cross-sectional study method, which measures PTSD symptoms only once (28). Therefore, to provide a possible explanation for study result heterogeneity, it is advisable to use a single cohort to perform longitudinal studies spanning multiple time points. Additionally, we hope to better reveal the transformation of associations between *ADCYAP1R1* and PTSD over time.

Furthermore, longitudinal designs are adept in concentrating on how the gene affects the course of symptoms (29, 30). Additionally, the children are in key period of emotion health development to strengthen foundation, especially when they survived disaster (11). But they are easy to suffer second-hand trauma, such as television images and non-professional aid (11). One study have reported that children have higher risk of PTSD after watching distressing media images (31). Therefore, it might be helpful to identify high risk children and provide strong support to develop intervention strategies through characterizing the PTSD symptom trajectories precisely.

However, a longitudinal study of the relationship between *ADCYAP1R1* and the course of PTSD symptoms has not been reported. Thus, we know little about how the genotype affects the trajectory of PTSD development after trauma exposure. A longitudinal study would aim to address these issues.

### Our study design

To examine the effect of *ADCYAP1R1* on PTSD in children and adolescents, we genotyped 1,017 participants who experienced the 2008 Wenchuan Earthquake in China. We also measured their PTSD symptoms at 2.5, 3.5, 4.5, and 5.5 years after the earthquake. We cautiously investigated (a) whether *ADCYAP1R1* genotypes had a significant main effect or gene–environment interaction effect on the course of PTSD symptoms and (b) whether the association between *ADCYAP1R1* and PTSD symptoms differed across four time points after trauma exposure.

## Materials and methods

### Participants and procedure

All samples were from Beichuan County, Sichuan Province, China. The residents of Beichuan suffered a serious earthquake in 2008. As for the limited aim power and young age range of sample, they were arranged to central place firstly. Later, in order to test the influence of earthquake in the health of local primary and secondary school students, the local education department developed four surveys at 2.5 years (Time 1), 3.5 (Time 2), 4.5 (Time 3), and 5.5 years (Time 4) after the earthquake.

The measurement procedure of our study is as follows, which has also been reported in our previous studies (29, 32–37): (a) assistants and teachers introduced the aim of the survey and obtained consent from students before each interview; (b) the students completed the survey questionnaire; (c) the two most affected secondary schools were selected for genotyping through collecting saliva samples at 6.5 years after the earthquake, and these clinical data were paired with the existing questionnaire data.

At Time 1,817 participants joined the study; Time 2 included 846 participants; Time 3 included 977 participants; and Time 4 included 1,099 participants. There were 1,039 participants surveyed twice or more between Time 1 and Time 4 and included in the following analysis referred to a previous longitude study (38). After we excluded 20 subjects with missing ethnicity information and 2 who were not successfully genotyped, the final sample was 1,017 participants, ranging from 7 to 11 years old (mean = 8.35 years, *SD* = 0.789). Self-reports of gender showed that slightly more than half of our participants were girls (54%), and the others were boys. Among the participants, 67% were of Qiang Chinese ethnicity, and the other 33% were of Han Chinese ethnicity. The excluded participants did not differ from the included ones in age, exposure, gender, or ethnicity. In the final sample, 206 (20.3%) participants were surveyed twice, 122 (12%) participants were surveyed three times, and 689 (67.7%) participants were surveyed four times. The number of completed surveys was not associated with gender, age, ethnicity, or exposure.

All surveys were conducted with informed consent from the students and their guardians. The research was approved by the Institutional Review Board of the Institute of Psychology, Chinese Academy of Sciences, Beijing, China. Our study is completely in compliance with national legislation and the Declaration of Helsinki.

### Measurement: Posttraumatic stress disorder symptoms and earthquake-related trauma exposure

PTSD symptoms were measured by the PTSD Reaction Index (PTSD-RI) of DSM-4 (Child Version) (39). The PTSD-RI is a self-report scale involving 20 items. Each item uses a 5-point Likert scale (from 0 = never to 4 = most of the time) to reflect PTSD symptoms according to the experience in past month. The Chinese version of the PTSD-RI has shown psychometric properties well in Chinese people (40). Compared to the PTSD-RI, the Chinese version excludes three items: two items that assess related features (i.e., fear of recurrence, trauma-related guilt) and one item that shows higher scores in restricted affect assessment. The PTSD-RI showed good reliability in studies (29).

Earthquake-related trauma exposure was assessed with a self-reported questionnaire. The participants were required to answer five yes (1) or no (0) questions about whether they had (a) experienced the death of a family member death because of trauma; (b) been injured; (c) seen someone injured; (d) seen buildings collapse; and/or (e) been exposed to the corpse of an earthquake victim. The level of trauma exposure was defined as the sum of scores for the 5 items. These five items were extensively used in our previous survey. Because the distribution of the total trauma score was not normal (Kolmogorov–Smirnov test, *p* < 0.001), as earlier studies suggested (41), we used a median split: a score of 0 or 1 was placed in the low-exposure group and coded as 0; a score of 2, 3, 4, or 5 was placed in the high-exposure group and coded as 1.

### Genotyping

Oragene (Canada) provided OG500 Kits to extract DNA. A custom-designed 2 × 48-Plex SNPscan™ Kit (Genesky Biotechnologies Inc., Shanghai, China) was used to perform the genotyping. For every participant, we used polymerase chain reaction (PCR) to amplify 10 ng DNA and conducted the following genotyping according to multiplex fluorescence PCR. In order to avoid genotyping errors, blind tests were performed. In the present study, the frequency of the rs2267735 genotype (CC: 30.8%, CG: 49.3%, GG: 19.9%) was in Hardy-Weinberg equilibrium (HWE).

### Statistical analysis

The association between the *ADCYAP1R1* polymorphism rs2267735 and the course of PTSD symptoms (including the main and gene-environment effects) was examined in all subjects, including girls and boys.

Latent growth models (LGMs) are a structural equation model (SEM) method that can well describe the changes within and between individuals over time. Thus, we decided to employ Mplus (version 7, city of Los Angeles, California, the United States) to conduct LGM to study PTSD symptoms. To examine the trajectory of PTSD symptoms between 2.5 and 5.5 years after the earthquake, we tested both linear and quadratic growth models. The models’ estimation method was full information maximum likelihood estimation with robust standard errors. The intercept was centered at the time point, 2.5 years after the earthquake. Evidence of acceptable fit was defined as a root mean square error of approximation (RMSEA) ≤ 0.08, standardized root mean square residual (SRMR) ≤ 0.08, comparative fit index (CFI) ≥ 0.9 and Tucker–Lewis index (TLI) ≥ 0.9 (42). Then, the main effect of gene was estimated in linear and quadratic growth models, with covariates of sex (0 for boys and 1 for girls; used in the analyses of all subjects only), age, ethnicity (0 for Han Chinese and 1 for Qiang Chinese), and trauma exposure (this last item is an environmental factor); the gene-environment effect was calculated later by adding interactive items to those two models. Genotypes were coded additively for the minor allele G (0 for CC, 1 for CG and 2 for GG). The results that had better model fits were selected for discussion. Furthermore, if the effect of G × E interaction was significant, the specific symptom course changes of G × E 0, G × E 1, and G × E 2 were compared using a three-group LGM. Finally, the main and G × E effects at each time point were calculated by centering the intercept of each time point within the LGM separately.

## Results

### Descriptive analyses

The descriptive results are present in Table 1. The severity of PTSD symptoms in girls was higher than that in boys (*p* < 0.05) over time. Significant correlations between ethnicity and PTSD were found at Times 1 (β = 0.108, *p* = 0.019), 2 (β = 0.098, *p* = 0.006), and 3 (β = 0.081, *p* = 0.014). The PTSD-RI scores of Qiang children were higher than those of Han children. The association between age and PTSD-RI scores was not significant at any time.

**TABLE 1:** The descriptive results of samples in our study.

		*M*	*SD*	Range
All	Age	8.35	0.789	7–11
	Environment	1.93	1.207	0–5
	PTSDRI at time 1	17.27	9.659	0–62
	PTSDRI at time 2	14.73	10.061	0–59
	PTSDRI at time 3	14.96	10.442	0–58
	PTSDRI at time 4	14.01	11.565	0–68
Male	Age	8.41	0.757	7–10
	Environment	1.93	1.183	0–5
	PTSDRI at time 1	16.72	9.388	0–49
	PTSDRI at time 2	13.00	9.053	0–50
	PTSDRI at time 3	13.40	10.309	0–52
	PTSDRI at time 4	12.70	11.744	0–68
Female	Age	8.30	0.813	7–11
	Environment	1.93	1.228	0–5
	(PTSDRI at time 1	17.74	9.869	0–62
	PTSDRI at time 2	16.20	10.634	0–59
	PTSDRI at time 3	16.29	10.381	0–58
	PTSDRI at time 4	15.14	11.299	0–62

### Latent growth analyses

#### Results in all subjects

To explore the trajectory of PTSD symptoms from Time 1 to Time 4, we tested linear and quadratic growth models. The linear growth model fit our data well [χ^2^ (df = 5, *N* = 1,017) = 29.578, RMSEA = 0.070, CFI = 0.961, TLI = 0.953, and SRMR = 0.050]. The quadratic growth mode did not fit well: [χ^2^ (df = 1, *N* = 1,017) = 18.456, RMSEA = 0.131, CFI = 0.972, TLI = 0.834, and SRMR = 0.025]. Thus, the course of PTSD symptom followed a linear pattern and the linear growth model was chosen for follow-up analysis. The intercept for PTSD symptoms, the indication of the PTSD-RI score of Time 1, was 16.558 (*p* < 0.001). The slope for symptoms, the indication of the change in PTSD-RI scores between consecutive time points, was –0.855 (*p* < 0.001). This suggests that PTSD symptoms significantly recovered from Time 1 to Time 4. The intercept and slope were not associated (*r* = –0.014, *p* = 0.911), which suggested the recovery rate was not associated with symptom severity at 2.5 years.

We used four models to examine the main and gene-environment interaction effects of genes on the course of PTSD symptoms. As for the main effect of rs2267735, the linear growth model fit our data well [χ^2^ (df = 15, *N* = 1,017) = 41.422, RMSEA = 0.042, CFI = 0.971, TLI = 0.949, and SRMR = 0.034]. The quadratic growth model also fit well: [χ^2^ (df = 6, *N* = 1,017) = 19.456, RMSEA = 0.047, CFI = 0.985, TLI = 0.935 and SRMR = 0.017]. However, the χ^2^/df value of the quadratic growth model is more than 3. Thus, the linear growth model was chosen for follow-up analysis. According to the analysis results, the gene did not significantly affect the intercept (β = 0.031, *p* = 0.496) or slope (β = 0.051, *p* = 0.346) of the course of PTSD symptoms. These results indicated that those with the CC genotype did not significantly exhibit higher PTSD symptom severity at 2.5 years or faster recovery rates than G allele carriers. Sex, ethnicity, and trauma exposure predicted the intercept. Regarding the gene-environment interaction (rs2267735 × trauma exposure) effect, the linear growth model fit our data well [χ^2^ (df = 17, *N* = 1,017) = 52.241, RMSEA = 0.045, CFI = 0.962, TLI = 0.933, and SRMR = 0.034]. The quadratic growth mode did not fit well: [χ^2^ (df = 17, *N* = 1,017) = 30.009, RMSEA = 0.057, CFI = 0.975, TLI = 0.894, and SRMR = 0.018]. The G × E effect was not significant for the intercept (β = –0.160, *p* = 0.083) or slope (β = 0.118, *p* = 0.287) (see Table 2).

**TABLE 2:** Main and gene–environment interaction effects of rs2267735 on the course of posttraumatic stress disorder symptoms between 2.5 and 5.5 years after the earthquake (across all subjects).

Predictor	B	SE	β	*P*
**Intercept**				
Gender	1.585	0.602	0.122	0.006
Ethnicity	1.491	0.625	0.108	0.019
Age	0.057	0.378	0.007	0.880
Environment	2.677	0.608	0.202	< 0.001
G	0.286	0.419	0.031	0.496
G × environment	–1.462	0.845	–0.160	0.083
**Slope**				
Gender	0.419	0.272	0.087	0.136
Ethnicity	–0.052	0.290	–0.010	0.857
Age	0.139	0.176	0.046	0.433
Environment	0.031	0.276	0.006	0.911
G	0.173	0.183	0.051	0.346
G × environment	0.396	0.370	0.118	0.287

The results at each time point indicated that the main and G × E (rs2267735 × trauma exposure) effects were not associated with PTSD symptoms at any of the four time points (Table 3).

**TABLE 3:** Main and gene–environment interaction effects of rs2267735 on the course of posttraumatic stress disorder symptoms at 2.5, 3.5, 4.5, and 5.5 years after the earthquake (across all subjects).

Predictor	B	SE	β	*P*
**Time 1 (2.5 years)**				
Gender	1.585	0.602	0.122	0.006
Ethnicity	1.491	0.625	0.108	0.019
Age	0.057	0.378	0.007	0.880
Environment	2.677	0.608	0.202	< 0.001
G	0.286	0.419	0.031	0.496
G × environment	–1.462	0.845	–0.160	0.083
**Time 2 (3.5 years)**				
Gender	2.004	0.510	0.145	< 0.001
Ethnicity	1.439	0.522	0.098	0.006
Age	0.196	0.315	0.022	0.531
Environment	2.707	0.514	0.193	< 0.001
G	0.459	0.353	0.047	0.195
G × environment	–1.066	0.711	–0.110	0.132
**Time 3 (4.5 years)**				
Gender	2.423	0.553	0.151	< 0.001
Ethnicity	1.387	0.569	0.081	0.014
Age	0.336	0.341	0.033	0.325
Environment	2.738	0.557	0.167	< 0.001
G	0.632	0.375	0.055	0.093
G × environment	–0.671	0.755	–0.059	0.373
**Time 4 (5.5 years)**				
Gender	2.841	0.707	0.147	< 0.001
Ethnicity	1.334	0.737	0.065	0.069
Age	0.475	0.443	0.039	0.285
Environment	2.769	0.714	0.141	< 0.001
G	0.805	0.474	0.059	0.089
G × environment	–0.276	0.953	–0.020	0.772

#### Results in girls

To explore the trajectory of PTSD symptoms from Time 1 to Time 4 in girls, we tested linear and quadratic growth models. The linear growth model fit our data well [χ^2^ (df = 5, *N* = 549) = 9.090, RMSEA = 0.039, CFI = 0.990, TLI = 0.988, and SRMR = 0.040]. The quadratic growth mode model did not fit well: [χ^2^ (df = 1, *N* = 549) = 6.437, RMSEA = 0.100, CFI = 0.987, TLI = 0.922, and SRMR = 0.020]. Thus, the course of PTSD symptom followed a linear pattern and the linear growth model was chosen for further analysis. The intercept for the course of PTSD symptoms, the indication of the PTSD-RI score of Time 1, was 17.319 (*p* < 0.001). The slope for symptoms, the indication of the change in PTSD-RI scores between consecutive time points, was –0.672 (*p* < 0.001). This suggests that PTSD symptoms significantly recovered from Time 1 to Time 4. The intercept and slope were not associated (*r* = –0.117, *p* = 0.420), which suggested the recovery rate was not associated with symptom severity at 2.5 years.

We used four models to examine the main and gene-environment interaction effects of genes on the course of PTSD symptoms. For the main effect of rs2267735, the linear growth model fit our data well [χ^2^ (df = 13, *N* = 549) = 11.942, RMSEA = 0.000, CFI = 1.00, TLI = 1.003, and SRMR = 0.030]. The quadratic growth mode also fit well: [χ^2^ (df = 5, *N* = 549) = 6.202, RMSEA = 0.021, CFI = 0.998, TLI = 0.990, and SRMR = 0.015], but worse than the linear growth model. Therefore, the linear growth model was chosen for follow-up analysis. According to the analysis results, the gene did not affect the intercept (β = 0.035, *p* = 0.555) or slope (β = 0.051, *p* = 0.482) of the course of PTSD symptoms. These results indicated that those with the CC genotype did not exhibit significantly higher PTSD symptom severity at 2.5 years or faster recovery rates than G allele carriers. Trauma exposure predicted the intercept. Regarding the gene-environment interaction (rs2267735 × trauma exposure) effect, the linear growth model fit our data well [χ^2^ (df = 15, *N* = 549) = 16.992, RMSEA = 0.016, CFI = 0.996, TLI = 0.994, and SRMR = 0.029]. The quadratic growth mode also fit well: [χ^2^ (df = 6, *N* = 549) = 11.379, RMSEA = 0.040, CFI = 0.990, TLI = 0.959, and SRMR = 0.016], but it was not better than the linear growth model. Thus, we chose the linear growth model. The G × E effect was significant for the intercept (β = –0.291, *p* = 0.013) but not the slope (β = 0.062, *p* = 0.674) (see Table 4).

**TABLE 4:** Main and gene–environment interaction effects of rs2267735 on the course of posttraumatic stress disorder symptoms between 2.5 and 5.5 years after the earthquake (in girls).

Predictor	B	SE	β	*P*
**Intercept**				
Ethnicity	1.254	0.915	0.082	0.181
Age	–0.121	0.528	–0.014	0.819
Environment	3.627	0.851	0.252	< 0.001
G	0.349	0.588	0.035	0.555
G × environment	–2.867	1.153	–0.291	0.013
**Slope**				
Ethnicity	0.031	0.390	0.006	0.938
Age	0.292	0.223	0.099	0.194
Environment	–0.349	0.361	–0.072	0.339
G	0.172	0.243	0.051	0.482
G × environment	0.205	0.486	0.062	0.674

**FIGURE 1:**
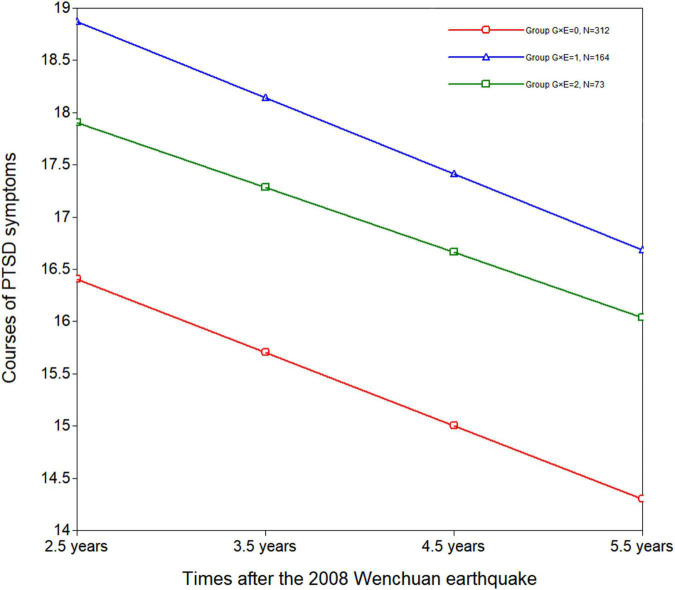
Courses of PTSD symptoms between 2.5 and 5.5 years after the 2008 Wenchuan earthquake by G × E (rs2267735 × trauma exposure) interaction in girls. 0: *n* = 312; G × E = 1: *n* = 164; G × E = 2: *n* = 73.

In order to assess the details of the G × E interaction, a three-group LGM was built. It was a very good fit for the data [χ^2^ (df = 15, *N* = 549) = 17.922, RMSEA = 0.033, CFI = 0.994, TLI = 0.992, and SRMR = 0.046]. The estimated course of PTSD symptoms by G × E is presented in Figure 1. According to the three-group LGM, the course of PTSD symptoms intercept was 16.406 (*p* < 0.001), and the slope was –0.701 (*p* = 0.003) for G × E = 0. For G × E = 1, the intercept was 18.871 (*p* < 0.001), and the slope was –0.730 (*p* = 0.026). For G × E = 2, the intercept was 17.902 (*p* < 0.001), and the slope was –0.621 (*p* = 0.178). The intercept and slope were not correlated in G × E = 0 (*r* = –0.225, *p* = 0.157), G × E = 1 (*r* = 0.334, *p* = 0.558) or G × E = 2 (*r* = –0.270, *p* = 0.319).

The results at each time point indicated that the G × E interaction (rs2267735 × trauma exposure) was associated with PTSD symptoms at Times 1, 2, and 3 in girls (*p* = 0.013, 0.005, and.013, respectively). See Table 5 for details.

**TABLE 5:** Main and gene–environment interaction effects of rs2267735 on the course of posttraumatic stress disorder symptoms at 2.5, 3.5, 4.5, and 5.5 years after the earthquake (in girls).

Predictor	B	SE	β	*P*
**Time 1 (2.5 years)**				
Ethnicity	1.254	0.915	0.082	0.181
Age	–0.121	0.528	–0.014	0.819
Environment	3.627	0.851	0.252	< 0.001
G	0.349	0.588	0.035	0.555
G × environment	–2.867	1.153	–0.291	0.013
**Time 2 (3.5 years)**				
Ethnicity	1.284	0.768	0.082	0.096
Age	0.171	0.440	0.019	0.698
Environment	3.278	0.724	0.223	< 0.001
G	0.520	0.497	0.051	0.300
G × environment	–2.662	0.963	–0.264	0.005
**Time 3 (4.5 years)**				
Ethnicity	1.315	0.805	0.075	0.101
Age	0.463	0.455	0.046	0.309
Environment	2.929	0.763	0.177	< 0.001
G	0.692	0.517	0.060	0.184
G × environment	–2.456	0.998	–0.217	0.013
**Time 4 (5.5 years)**				
Ethnicity	1.345	1.005	0.065	0.180
Age	0.754	0.566	0.064	0.181
Environment	2.580	0.949	0.133	0.006
G	0.864	0.636	0.064	0.178
G × environment	–2.250	1.240	–0.169	0.068

#### Results in boys

None of the LGMs for boys fit well, but we have still listed the results here.

In order to explore the trajectory of PTSD symptoms from Time 1 to Time 4 in boys, we tested linear and quadratic growth models. The linear growth model did not fit our data very well [χ^2^ (df = 5, *N* = 468) = 28.532, RMSEA = 0.100, CFI = 0.895, TLI = 0.873, and SRMR = 0.064]. The quadratic growth model did not fit well: [χ^2^ (df = 1, *N* = 468) = 13.308, RMSEA = 0.162, CFI = 0.945, TLI = 0.669, and SRMR = 0.032]. In LGM, the intercept for course of PTSD symptoms, the indication of the PTSD-RI scores at Time 1, was 15.548 (*p* < 0.001). The slope for symptoms, the indication of the change in PTSD-RI between consecutive time points, was –1.040 (*p* < 0.001). This suggests that PTSD symptoms significantly recovered from Time 1 to Time 4. The intercept and slope were not associated (*r* = 0.104, *p* = 0.651), which suggested the recovery rate was not associated with symptom severity at 2.5 years.

We used four models to examine the main and gene-environment interaction effects of genes on the course of PTSD symptoms. As for the main effect of rs2267735, the linear growth model did not fit our data well [χ^2^ (df = 13, *N* = 468) = 40.761, RMSEA = 0.068, CFI = 0.912, TLI = 0.851, and SRMR = 0.046]. The quadratic growth model also did not fit: [χ^2^ (df = 5, *N* = 468) = 14.754, RMSEA = 0.065, CFI = 0.969, TLI = 0.864, and SRMR = 0.022]. In LGM, the gene did not significantly affect the intercept (β = 0.021, *p* = 0.783) and slope (β = 0.052, *p* = 0.523) of the course of PTSD symptoms. The results indicated that those with the CC genotype did not exhibit higher PTSD symptom severity at 2.5 years or faster recovery rates than G allele carriers. Regarding the gene-environment interaction (rs2267735 × trauma exposure) effect, the linear growth model did not fit our data well [χ^2^ (df = 15, *N* = 468) = 47.149, RMSEA = 0.068, CFI = 0.902, TLI = 0.830, and SRMR = 0.047]. The quadratic growth model did not fit well: [χ^2^ (df = 6, *N* = 468) = 20.092, RMSEA = 0.071, CFI = 0.957, TLI = 0.814, and SRMR = 0.022]. In the LGM, the G × E effect was not significant for the intercept (β = 0.035, *p* = 0.819) or slope (β = 0.178, *p* = 0.278) (see Table 6).

**TABLE 6:** Main and gene–environment interaction effects of rs2267735 on the course of posttraumatic stress disorder symptoms between 2.5 and 5.5 years after the earthquake (in boys).

Predictor	B	SE	β	*P*
**Intercept**				
Ethnicity	1.550	0.860	0.133	0.062
Age	0.177	0.526	0.024	0.736
Environment	1.466	0.865	0.129	0.088
G	0.164	0.593	0.021	0.783
G × environment	0.281	1.229	0.035	0.819
**Slope**				
Ethnicity	–0.062	0.440	–0.012	0.888
Age	–0.023	0.277	–0.007	0.935
Environment	0.484	0.422	0.099	0.255
G	0.178	0.280	0.052	0.523
G × environment	0.606	0.564	0.178	0.278

The results of each time point indicated that the main and G × E (rs2267735 × trauma exposure) effects were not associated with PTSD symptoms at any of the four time points (Table 7).

**TABLE 7:** Main and gene–environment interaction effects of rs2267735 on the course of posttraumatic stress disorder symptoms at 2.5, 3.5, 4.5, and 5.5 years after the earthquake (in boys).

Predictor	B	SE	β	*P*
**Time 1 (2.5 years)**				
Ethnicity	1.550	0.860	0.133	0.062
Age	0.177	0.526	0.024	0.736
Environment	1.466	0.865	0.129	0.088
G	0.164	0.593	0.021	0.783
G × environment	0.281	1.229	0.035	0.819
**Time 2 (3.5 years)**				
Ethnicity	1.488	0.702	0.114	0.030
Age	0.154	0.440	0.019	0.725
Environment	1.950	0.725	0.153	0.006
G	0.342	0.501	0.038	0.494
G × environment	0.887	1.037	0.099	0.392
**Time 3 (4.5 years)**				
Ethnicity	1.427	0.797	0.090	0.070
Age	0.131	0.514	0.013	0.798
Environment	2.434	0.811	0.156	0.002
G	0.520	0.554	0.048	0.344
G × environment	1.494	1.131	0.136	0.187
**Time 4 (5.5 years)**				
Ethnicity	1.365	1.080	0.069	0.204
Age	0.109	0.699	0.009	0.877
Environment	2.917	1.071	0.151	0.006
G	0.699	0.721	0.052	0.328
G × environment	2.100	1.456	0.155	0.149

## Discussion

### Summary of results

This longitudinal study analyzed 1,017 children and adolescents who survived the 2008 Wenchuan earthquake. Regarding cutoff scores on the PTSD-RI, participants suffered mild PTSD symptoms between 2.5 and 5.5 years after the earthquake (39). Although the scores fell short of the cutoff threshold required for diagnosis, subthreshold levels of PTSD are still clinically meaningful and can indicate marked impairment (43).

This study successfully tested the main and gene-environment effects of the *ADCYAP1R1* polymorphism rs2267735 on girl PTSD symptoms longitudinally after the earthquake. The *ADCYAP1R1*-environment interaction was significantly correlated with the course of PTSD symptoms in girls between 2.5 and 4.5 years after disaster.

Previous studies have reported that, among highly traumatized females, CC genotype carriers had higher PTSD symptom scores (24) than G allele carriers. In our longitudinal study, the G × E results in high trauma load girls agreed with these findings. Additionally, as the intercept was not significantly correlated with the slope, it appears that the baseline symptom severity did not affect the PTSD recovery rate. Moreover, our longitudinal study reported that the effect of the *ADCYAP1R1*-environment interaction on PTSD symptoms might involve changes in symptoms over time. This finding confirmed the opinion that genetic factors could also influence the course of PTSD symptoms (30, 44, 45). Obviously, additional longitudinal genetic studies are needed.

### Details of the gene-environment interaction effect on girls

To explore the details of the G × E effect on girls, we divided girls into two groups according to different trauma exposure levels (E = 0 and E = 1) and performed LGM analyses. In the low-exposure group (E = 0), the linear LGM fit the data well, and the main effect was associated with intercept (β = 0.245 and *p* = 0.007) but not slope (*p* = 0.925). The main effect also correlated with PTSD symptoms at all four time points. At each time point, compared with G allele carriers, subjects with the CC genotype had reduced PTSD symptom scores. The three-group LGM did not fit the data well. More details are provided in Supplementary Tables 1, 2 and Supplementary Figure 1. In the high-exposure group (E = 1), the linear LGM did not fit the data well, while the three-group LGM fit the data well. According to the results of three-group LGM, compared with G allele carriers, the CC genotype had slightly higher PTSD symptom scores and a faster PTSD symptom recovery rate. These findings were not statistically strong, possibly due to the relatively modest sample size. Further details are provided in Supplementary Tables 3, 4 and Supplementary Figure 2.

### Interpretation

Consistent with results from previous cross-sectional studies [i.e., Almli et al. (24) repeated the finding of Ressler et al. (22) that higher trauma could predict PTSD symptom severity in carriers of the “C” allele; Uddin et al. (27) found the childhood maltreatment trauma positively predicted PTSD symptoms most strongly in CC genotype carriers], we also found that rs2267735 × trauma exposure was associated with PTSD symptoms in girls between 2.5 and 4.5 years after the earthquake. It might be because the SNP rs2267738 locates in elements which response to putative estrogen, so its association was in a female-specific model (46–52). Meanwhile, the environment affairs could also increase the risk of PTSD through epigenetic mechanisms (53). But this effect was no longer significant at the fourth time point. A longitude study showed that compared with 8–18 years old children who suffered accidental injury only 3 months, 11–22 years old children after 2–4 years of accident performed lower prevalence of PTSD (54). Thus, we assumed the influence of the earthquake perhaps weakened with the development of children and it would be interesting to use much longer-term follow-up study in children to address this issue in the future.

Contrary to some studies (22, 23, 55), our results did not show a main effect of the *ADCYAP1R1* polymorphism rs2267735. However, some previous research also found the significance only in gene-environment interaction effect only, which were similar with us (24, 27). Regarding these findings, it is more worth mentioning that genes affect symptoms more readily by interacting with the environment than by acting in isolation. Thus, it demonstrated that we could further explore how the environment affects the *ADCYAP1R1* to influence symptoms.

Our sample is unique. The participants are all from China, not Western countries, in contrast to the subjects of previous Western studies. In addition, our study tracks the sample over a long period. Most other studies collected responses from participants over a span of less than 3 years. However, our participants were measured four times, the last of which was 5.5 years after the earthquake.

### Limitations

The current study has some limitations. The first assessment for participants was 2.5 years after the earthquake, and it did not capture PTSD symptoms immediately following the disaster. Furthermore, owing to the limited number of questions, the assessment of trauma exposure is not very precise and does not adequately capture subtle differences in trauma exposure. In addition, our study used only a single type of traumatic event, an earthquake; similar studies should be performed for other trauma types. Finally, the ethnicity effects showed significant at 2.5, 3.5, and 5.5 years after earthquake across all subjects and weaker than gender. But the number of Qiang and Han had a big gap, thus we could control the ethnic sample in the future and then detect the main effect of ethnicity.

## Conclusion

The current study is the first longitudinal study to investigate the association between the *ADCYAP1R1* polymorphism rs2267735 and the course of PTSD symptoms in children and adolescents. According to measures of PTSD symptoms between 2.5 and 5.5 years after trauma exposure, there was a significant association between gene-environment interaction and the course of PTSD symptoms, which provides a fresh perspective regarding the effect of this gene on PTSD.

## Data availability statement

The original contributions presented in this study are included in the article/Supplementary material, further inquiries can be directed to the corresponding author/s.

## Ethics statement

The studies involving human participants were reviewed and approved by Institutional Review Board of the Institute of Psychology, Chinese Academy of Sciences, Beijing, China. Written informed consent to participate in this study was provided by the participants or their legal guardian/next of kin.

## Author contributions

LW and KZ conceived and designed the overall study. LW collected the samples. KZ, JZ, YZ, and GZ performed the statistical analysis. CC and HY performed the genotyping. PL and SL took part in sample collection. JZ, KZ, and LW wrote the manuscript. GL and RF helped to revise the manuscript. All authors read and approved the final version of the manuscript.
