# Supplementary material for: The main effect and gene-environment interaction effect of the ADCYAP1R1 polymorphism rs2267735 on the course of posttraumatic stress disorder symptoms—A longitudinal analysis
Source: Front Psychiatry. 2022 Oct 28;13:1032837. doi: 10.3389/fpsyt.2022.1032837 (PMC9650374; doi:10.3389/fpsyt.2022.1032837)
Supplement: Supplementary file 1 [file Data_Sheet_1.docx]

Supplementary Material

# The relationship between genes and PTSD symptoms in different exposure groups (in girls)

## Low-exposure group (E=0)

We used a linear growth model to examine the main effect of genes on PTSD symptoms. The model fit our data well (*χ*^2^ (df = 11, *N* = 221) = 16.955, RMSEA = 0.049, CFI = 0.947, TLI = 0.957 and SRMR = 0.049). The genotype significantly affected the intercept (𝛽 = 0.245, *p* = .007) but not the slope (𝛽 = -0.009, *p* = .925) of the course of PTSD symptoms. The results indicated that those with the CC genotype had lesser initial PTSD symptoms than G allele carriers (see **Supplementary Table 1**).

The results at each time point indicated that the main (rs2267735) effect was associated with PTSD symptoms at all four time points (**Supplementary Table 2**).

## High-exposure group (E=1)

We used a linear growth model to examine the main effect of genes on PTSD symptoms. The model fit our data well: *χ*^2^ (df = 11, *N* = 328) = 9.171, RMSEA = 0.000, CFI = 1.000, TLI = 1.010 and SRMR = 0.030. The genotype did not significantly affect the intercept (𝛽 = -0.085, *p* = .273) or slope (𝛽 = 0.072, *p* = .471) of the course of PTSD symptoms (see **Supplementary Table 3**).

The results of each time point indicated that the main (rs2267735) effect was not associated with PTSD symptoms at any of the four time points (**Supplementary Table 4**).

# The three-group latent growth model (for girls)

## Low-exposure group (E=0)

In order to assess the details of G×E interaction, a three-group latent growth model (LGM) was built. It was a poor fit for the data (*χ*^2^ (df = 15, *N* = 221) = 26.883, RMSEA = 0.104, CFI = 0.945, TLI = 0.933 and SRMR = 0.075). The estimated results concerning the course of PTSD symptoms are presented in **Supplementary Figure 1**. According to the three-group LGM, the intercept for the course of PTSD symptoms was 13.559 (*p* < .001), and the slope was -0.541 (*p* = .211) for CC homozygotes. For CG, the intercept was 15.204 (*p* < .001), and the slope was -0.555 (*p* = .225). For GG, the intercept was 18.014 (*p* < .001), and the slope was -0.438 (*p* = .388). The intercept and slope were not correlated in either CC carriers (*r* = -0.237, *p* = .441), CG carriers (*r* = -0.282, *p* = .294) or GG carriers (*r* = 999.000, *p* = .317).

## High-exposure group (E=1)

Because the association between PTSD and genotype in the high trauma load group is always interesting, we still examined the details of G×E interaction. A three-group LGM was built. It was a very good fit for the data: *χ*^2^ (df = 15, *N* =328) = 15.017, RMSEA = 0.003, CFI = 1.00, TLI = 1.00, SRMR = 0.054. The estimated results concerning the course of PTSD symptoms are presented in **Supplementary Figure 2**. According to the three-group LGM, the intercept for the course of PTSD symptoms was 19.461 (*p* < .001), and the slope was -1.007 (*p* = .018) for CC homozygotes. For the CG allele, the intercept was 18.871 (*p* < .001), and the slope was −0.930 (*p* = .026). For the GG allele, the intercept was 17.903 (*p* < .001), and the slope was −0.622 (*p* = .178). The intercept and slope were not correlated in either CC allele carriers (*r* = -0.064, *p* = .875), CG homozygotes (*r* = 0.333, *p* = .584) or GG homozygotes (*r* = -0.270, *p* = .319).

# Supplementary Figures and Tables

**Supplementary Table 1** Main effect of rs2267735 on the course of posttraumatic stress disorder symptoms between 2.5 and 5.5 years after the earthquake (in girls). Note: E=0.

| **Predictor** | **B** | **SE** | ***β*** | ***P*** |
| --- | --- | --- | --- | --- |
| Intercept |  |  |  |  |
| Ethnicity | 0.801 | 1.301 | 0.058 | 0.539 |
| Age | -0.518 | 0.769 | -0.064 | 0.501 |
| G | 2.295 | 0.854 | 0.245 | **0.007** |
| Slope |  |  |  |  |
| Ethnicity | 0.304 | 0.595 | 0.059 | 0.609 |
| Age | 0.299 | 0.345 | 0.098 | 0.396 |
| G | -0.033 | 0.355 | -0.009 | 0.925 |

**Supplementary Table 2** Main effect of rs2267735 on the course of posttraumatic stress disorder symptoms at 2.5, 3.5, 4.5 and 5.5 years after the earthquake (in girls). Note: E=0.

| **Predictor** | **B** | **SE** | ***β*** | ***P*** |
| --- | --- | --- | --- | --- |
| Time 1 (2.5 years) |  |  |  |  |
| Ethnicity | 0.801 | 1.301 | 0.058 | 0.539 |
| Age | -0.518 | 0.769 | -0.064 | 0.501 |
| G | 2.295 | 0.854 | 0.245 | **0.007** |
| Time 2 (3.5 years) |  |  |  |  |
| Ethnicity | 1.105 | 0.595 | 0.082 | 0.305 |
| Age | -0.219 | 0.345 | -0.027 | 0.725 |
| G | -0.033 | 0.355 | 0.244 | **0.001** |
| Time 3 (4.5 years) |  |  |  |  |
| Ethnicity | 1.410 | 1.166 | 0.092 | 0.235 |
| Age | 0.081 | 0.649 | 0.009 | 0.901 |
| G | 2.228 | 0.695 | 0.214 | **0.002** |
| Time 4 (5.5 years) |  |  |  |  |
| Ethnicity | 1.714 | 1.501 | 0.094 | 0.268 |
| Age | 0.380 | 0.834 | 0.035 | 0.649 |
| G | 2.195 | 0.858 | 0.176 | **0.012** |

**Supplementary Table 3** Main effect of rs2267735 on the course of posttraumatic stress disorder symptoms between 2.5 and 5.5 years after the earthquake (in girls). Note: E=1.

| **Predictor** | **B** | **SE** | ***β*** | ***P*** |
| --- | --- | --- | --- | --- |
| Intercept |  |  |  |  |
| Ethnicity | 1.822 | 1.261 | 0.117 | 0.173 |
| Age | 0.183 | 0.721 | 0.021 | 0.800 |
| G | -0.836 | 0.768 | -0.085 | 0.273 |
| Slope |  |  |  |  |
| Ethnicity | -0.126 | 0.506 | -0.025 | 0.805 |
| Age | 0.252 | 0.285 | 0.088 | 0.377 |
| G | 0.234 | 0.318 | 0.072 | 0.471 |

**Supplementary Table 4** Main effect of rs2267735 on the course of posttraumatic stress disorder symptoms at 2.5, 3.5, 4.5 and 5.5 years after the earthquake (in girls). Note: E=1.

| **Predictor** | **B** | **SE** | ***β*** | ***P*** |
| --- | --- | --- | --- | --- |
| Time 1 (2.5 years) |  |  |  |  |
| Ethnicity | 1.822 | 1.261 | 0.117 | 0.173 |
| Age | 0.183 | 0.721 | 0.021 | 0.800 |
| G | -0.836 | 0.768 | -0.085 | 0.273 |
| Time 2 (3.5 years) |  |  |  |  |
| Ethnicity | 1.696 | 1.062 | 0.103 | 0.114 |
| Age | 0.435 | 0.614 | 0.047 | 0.477 |
| G | -0.601 | 0.662 | -0.058 | 0.359 |
| Time 3 (4.5 years) |  |  |  |  |
| Ethnicity | 1.570 | 1.086 | 0.084 | 0.141 |
| Age | 0.687 | 0.629 | 0.066 | 0.272 |
| G | -0.367 | 0.700 | -0.031 | 0.598 |
| Time 4 (5.5 years) |  |  |  |  |
| Ethnicity | 1.443 | 1.320 | 0.066 | 0.265 |
| Age | 0.939 | 0.759 | 0.077 | 0.213 |
| G | -0.132 | 0.863 | -0.010 | 0.879 |

## Supplementary Figures


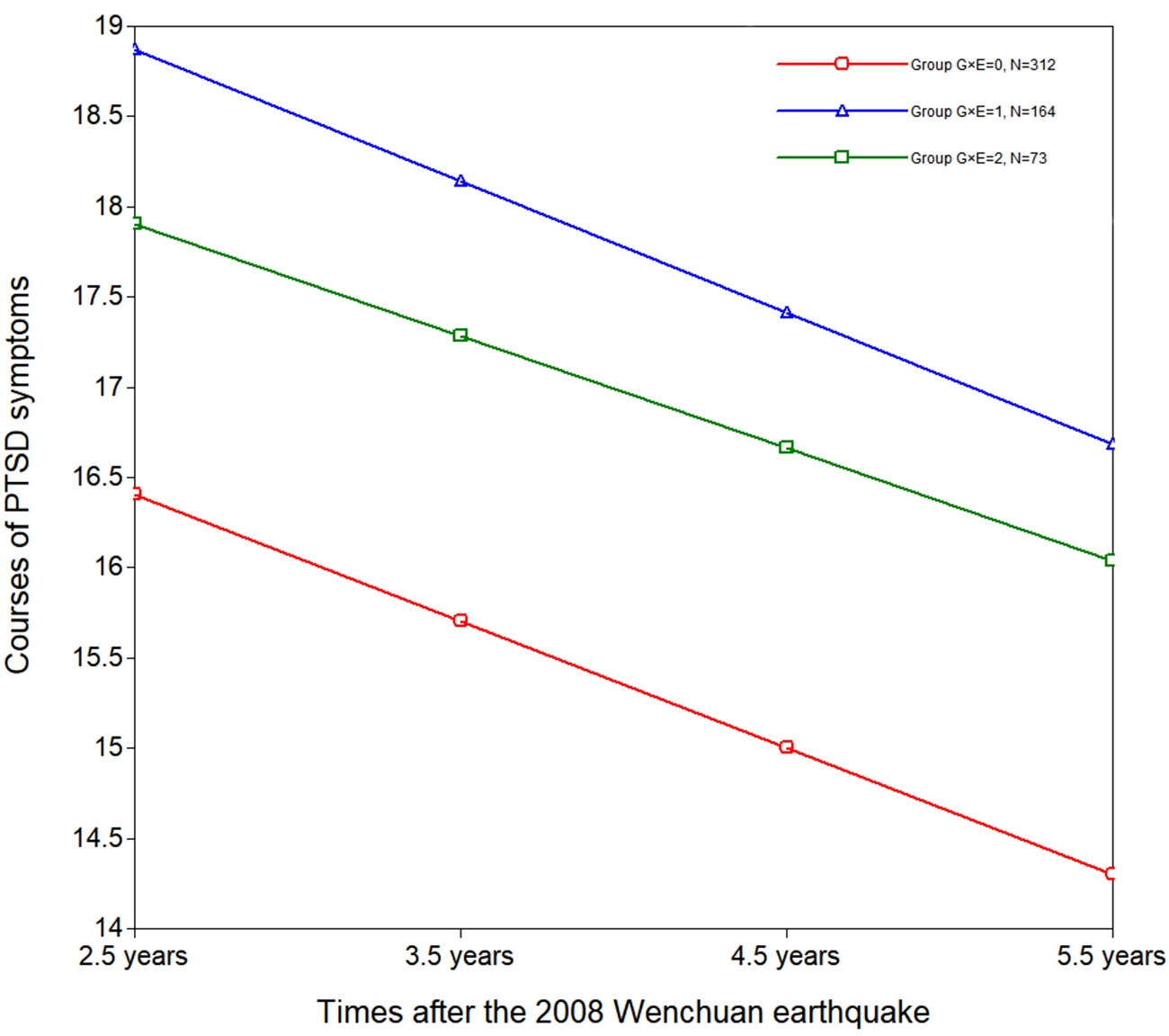


**Supplementary Figure 1** Courses of PTSD symptoms between 2.5 and 5.5 years after the 2008 Wenchuan earthquake by rs2267735 genotype in girls. Note: E=0; GG: *n* = 38; CG: *n* = 105; CC: *n* = 78.


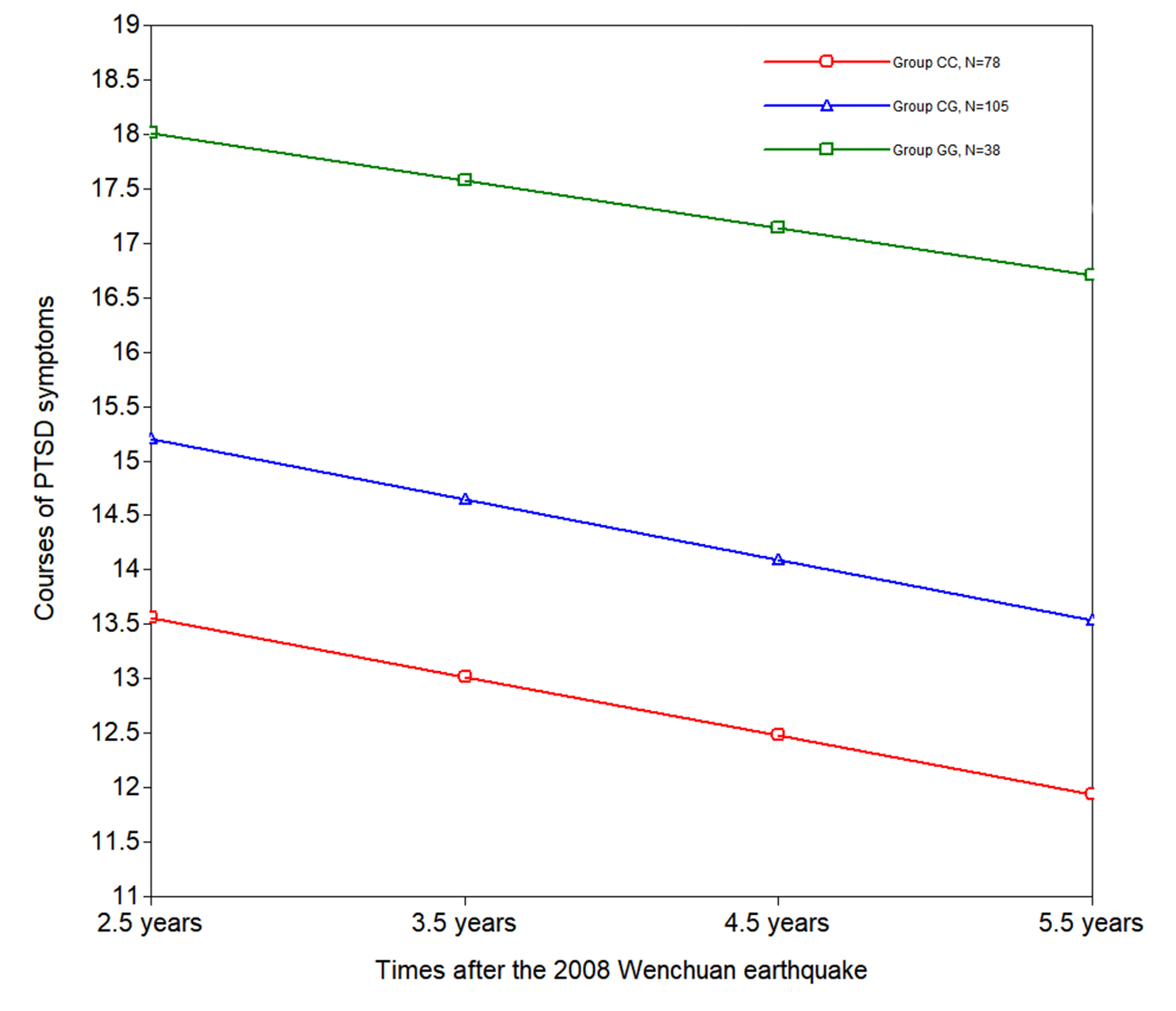


**Supplementary Figure 2** Courses of PTSD symptoms between 2.5 and 5.5 years after the 2008 Wenchuan earthquake by rs2267735 genotype in girls. Note: E=1; GG: *n* = 73; CG: *n* = 164; CC: *n* = 91.
